# Impact of Corn Oil on Biochemical and Neurochemical Markers in an Animal Model of Alzheimer’s Disease

**DOI:** 10.3390/neurolint18090176

**Published:** 2026-09-17

**Authors:** Jadyellen Rondon Silva, Maria Elisa Fonseca Oliveira, Lira Kamila Palacios Campos, Marcos Jose Jacinto, Anderson Oliveira Souza

**Affiliations:** 1 Mitochondrial Metabolism and Neurotoxicology Laboratory, Department of Chemistry, Institute of Chemistry, Federal University of Mato Grosso, Cuiabá 79070-900, Brazil; jrondon586@gmail.com (J.R.S.); oliveira.mariaelisa01@gmail.com (M.E.F.O.); lirapalaciost@gmail.com (L.K.P.C.); 2 Department of Chemistry, Institute of Chemistry, Federal University of Mato Grosso, Cuiabá 79070-900, Brazil; marcjrdt@icloud.com

**Keywords:** *Drosophila melanogaster*, functional food, biochemical and neurochemical markers, Alzheimer’s disease

## Abstract

Background: The ingestion of polyunsaturated fatty acids is vital for brain health, supporting cognitive development and helping to prevent chronic diseases, including neurodegenerative processes. Objective: This study aimed to investigate the effects of corn oil on the biochemical and neurochemical parameters of *Drosophila melanogaster* expressing human amyloid precursor protein β42 (APP-β_42_). Methods: The flies were fed a diet supplemented with 37.8 mg/mL of corn oil from the larval stage until adulthood. Results: A diet supplemented with corn oil induced significant changes in biochemical markers, such as a decrease in head cholesterol levels (*p* < 0.001); decreased catalase activity and hydrogen peroxide levels in the heads (*p* < 0.0001) and thoracic muscles (*p* < 0.0001); reduced glutathione levels in the heads (*p* < 0.0001) and muscles (*p* < 0.01); and reduced formazan production (*p* < 0.01) and citrate synthase (CS) activity (*p* < 0.0001) in the head. However, in thoracic muscle, ingestion of corn oil led to an increase in formazan production (*p* < 0.01) and no significant change in CS activity. Lactate levels decreased in the heads (*p* < 0.0001) and thoraces (*p* < 0.001) after flies were fed corn oil. Finally, ingestion of corn oil resulted in a significant rise in acetylcholinesterase activity in the heads (*p* < 0.001) and thoracic muscles (*p* < 0.01). Conclusions: These results suggest that consuming corn oil reduces the oxidative stress typical of an Alzheimer’s disease model by enhancing antioxidant defenses.

## 1. Introduction

Alzheimer’s disease (AD) is the primary cause of dementia worldwide, characterized by memory loss, language difficulties, impaired spatial orientation, and other significant cognitive declines, leading to decreased independence and quality of life [1]. Several hypotheses aim to explain its underlying mechanisms. One prominent theory is the amyloid cascade hypothesis, which involves the extracellular buildup of β-amyloid peptide plaques (Aβ) through APP metabolism [2]. Another is the tau hyperphosphorylation hypothesis, where neurofibrillary tangles composed of hyperphosphorylated tau protein are observed [3]. Additionally, evidence shows that chronic inflammation, oxidative stress, and metabolic alterations also significantly contribute to disease progression [4].

Diagnosis is typically confirmed through testing, followed by the initiation of treatment [5]. Currently, treatments mainly provide symptomatic relief, aiming to reduce cognitive and behavioral symptoms rather than halt the progression of the disease. Common medications include acetylcholinesterase inhibitors such as donepezil, rivastigmine, and galantamine. Memantine, an N-methyl-D-aspartate (NMDA) receptor antagonist, is also used [6]. Although new therapies are approved annually in various countries, their actual effectiveness remains a matter of debate, as these drugs do not stop long-term disease progression and can cause side effects [7].

The primary existing drugs for AD are acetylcholinesterase inhibitors, which work by preventing the degradation of acetylcholine and supporting cholinergic transmission [8]. Based on the inflammatory aspect of AD, steroidal and non-steroidal anti-inflammatory drugs have also been explored as treatment options. However, they generally do not show satisfactory results and are associated with hepatotoxicity [9]. Additionally, monoclonal antibodies are believed to activate microglia to perform phagocytosis, which could prevent the formation of new amyloid plaques and slow disease progression [10].

Although these drugs help slow disease progression, they all have side effects. Acetylcholinesterase inhibitors often cause gastrointestinal issues such as nausea, vomiting, and diarrhea, as well as cardiac side effects [11]. Conversely, newer monoclonal antibodies carry a significant risk of amyloid-related imaging abnormalities (ARIAs), which may manifest as cerebral edema or microhemorrhages and require careful monitoring [12].

Studies indicate that non-pharmacological approaches using functional foods significantly aid in managing chronic diseases, such as neurodegenerative conditions [13,14]. These foods are abundant in bioactive substances such as omega-3 fatty acids, probiotics, prebiotics, postbiotics, polyphenols, flavonoids, carotenes, lycopene, folic acid, inositol, glycolipids, phytosterols, phosphatidylcholine, oleic acid, and specific antimicrobial peptides that can enhance metabolic health [14].

Corn oil is a vegetable oil widely used in the diet; its composition is rich in polyunsaturated fatty acids, especially linoleic acid (omega-6) and oleic acid (omega-9). When incorporated into the usual diet in moderation, it helps improve the plasma lipid profile, thereby reducing cholesterol [15]. Other components of corn oil, such as tocopherols and phytosterols, may contribute to antioxidant activity, protecting lipid membranes against oxidative peroxidation [16]. In this study, we aimed to investigate the effects of corn oil ingestion on biochemical and neurochemical markers in *Drosophila melanogaster* expressing APP-β_42_.

## 2. Materials and Methods

### 2.1. Fly Strain and Rearing

All experiments in this study used mutant fly lines sourced from the Bloomington Drosophila Stock Center (BDSC). The flies were maintained at 25 ± 1 °C on a standard diet composed of cornmeal (6.5% *m*/*v*), agar (CAS no. 9002-18-00) (1.0% *m*/*v*), yeast (6.5%), and Nipagin (CAS no. 99-76-3) (3.0% *v*/*v*) [17,18] or supplemented with corn oil (Bunge Ltd.a, Guaíra, Brazil) [13]. A standard diet composed of cornmeal (75% carbohydrates, 6.2% protein, and 1.5% lipids) and yeast (42% carbohydrates, 42% protein, and 6% lipids) represents 47.9 kcal. Additionally, a standard diet supplemented with corn oil (92% lipids) provides 48.61 kcal to the flies [19].

#### 2.1.1. Phenotypic Expression

The line expressing amyloid precursor protein–β-amyloid peptide 42 (APP-β_42_) was cultured at 29 ± 1 °C and was created by recombining the APP-β_42_ transgene under the control of the upstream activation sequence (UAS)-APP-β_42_ with the glass multimer reporter Gal4 driver (GMR-Gal4) (BDSC 1104) on chromosome II, followed by crossing those flies with flies carrying UAS-APP-β_42_ (BDSC 33773) on chromosome II (Figure S1).

#### 2.1.2. Exposure to a Diet Supplemented with Corn Oil

Both male and female flies were maintained under the experimental dietary conditions; however, only females were used for biochemical analyses because of their greater metabolic sensitivity to dietary changes. Males were maintained together with females during the adult exposure period to support normal feeding behavior. For the dietary treatment, adult mutant (GMR-Gal4; UAS-APP-β_42_) flies were allowed to oviposit for 3 h directly on either (1) a standard diet or (2) a standard diet supplemented with corn oil at a final concentration of 37.8 mg/mL, based on the experimental conditions previously established in our published study [19]. After the oviposition period, the parental flies were removed, and the offspring developed under their respective experimental conditions. Following adult eclosion, the flies continued to receive the corresponding standard or corn oil-supplemented diet for an additional 5 days.

At 5 days post-eclosion, female flies were collected, and their heads and thoraces were dissected separately for subsequent biochemical analyses. For each experimental group, three independent biological replicates were prepared. Each biological replicate consisted of 100 female flies, with heads and thoraces pooled separately, resulting in one pool of 100 heads and one pool of 100 thoraces per biological replicate.

The pooled tissues were homogenized with tungsten carbide beads (cat. no. 69997, Qiagen) in a 0.9% NaCl solution (pH 7.0; CAS no. 7647-14-5) containing a protease inhibitor cocktail diluted 1:200 (CAS no. 66701-25-5). Homogenates were centrifuged at 10,000× *g* for 10 min at 4 °C. The supernatants were collected and stored at −20 °C until analysis.

### 2.2. Smurf Test

Flies aged 14 days were subjected to starvation for 2 h and were subsequently exposed for 4 h to a diet containing sucrose (CAS no. 57-50-1) (5.0% *m*/*v*), agar (1.0% *m*/*v*), and brilliant blue (CAS no. 3844-45-9) (2.5% *m*/*v*) [20]. Then, the flies were anesthetized with FlyNap, and the dye ingestion was recorded through images (Figure S2).

#### Total Cholesterol

Homogenates of mutant *D. melanogaster* heads and thoraces were used to determine cholesterol levels using a kit (Bioclin, Brazil, cat. no. #K083).

### 2.3. Reduced Glutathione (GSH) Levels

Heads and thoraces of mutant flies were homogenized in a 100 mM sodium phosphate buffer (pH 7.4) with 6 mM EDTA (CAS no. 60-00-4) and 1 mg/mL OPT (CAS no. 643-79-8) for 15 min [21]. Glutathione levels were then measured spectrophotometrically at 420 nm using a Varian Cary 50MPR spectrophotometer (Varian Ltd., Melbourne, Australia).

#### Lactate Content

Lactate levels were determined using the lactate oxidase method following the manufacturer’s instructions (Labtest, Brazil, cat. no. 138-1/50). Absorbance was measured spectrophotometrically at 550 nm [22] using a Varian Cary 50MPR spectrophotometer (Varian Ltd., Melbourne, Australia). The lactate concentration was reported in mg/dL, normalized to the total protein content in the sample.

### 2.4. Catalase (CAT) Activity

Head and thorax homogenate samples were added to a quartz cuvette with a 50 mM potassium phosphate buffer (pH 7.0) as described by Aebi (1984) [23]. The reaction was started by adding 0.3 M hydrogen peroxide (H_2_O_2_), and absorbance was measured at 240 nm using a Varian Cary 50MPR spectrophotometer (Varian Ltd., Melbourne, Australia). Enzyme activity was determined using the molar extinction coefficient of H_2_O_2_ and expressed as mU/min/mg of protein.

#### Hydrogen Peroxide (H_2_O_2_) Production

Ferrous oxidation with xylenol orange (FOX) involves using a solution containing 0.1 mM xylenol orange (CAS no. 3618-43-7), 0.25 mM ferrous ammonium sulfate (CAS no. 7783-85-9), and 100 mM sorbitol (CAS no. 50-70-4). A 10 μL sample was combined with 190 μL of FOX reagent, mixed thoroughly, and incubated at room temperature for at least 30 min [24]. The resulting reaction’s optical density was then measured at 560 nm with a Varian Cary 50MPR spectrophotometer (Cary 50MPR, Varian Ltd., Melbourne, Australia).

### 2.5. Citrate Synthase (CS) Activity

Citrate synthase activity was initiated by adding 0.01 mg of protein to approximately 170 μL of Tris (200 mM, pH 8.0) buffer containing Triton X-100 (CAS no. 9036-19-5) 0.2% (*v*/*v*), 10 mM acetyl-CoA (CAS no. 32140-51-5), 1 mM 5’5’-dithiobis-2-nitrobenzoic acid (DTNB) (CAS no. 69-78-3), and 10 mM oxaloacetate (CAS no. 328-42-7) [25]. The CoA-SH produced by CS reduces DTNB to TNB, a reaction that can be monitored. CS activity was assessed by measuring the rate of TNB formation spectrophotometrically at 412 nm, following Srere (1969) [26], using a Varian Cary 50MPR spectrophotometer (Varian Ltd., Melbourne, Australia).

#### Formazan Production

Formazan production was analyzed using 30 μL of MTT (1.25 mM; CAS no. 298-93-1), 20 μL of pyrogallol (10 mM; CAS no. 87-66-1), and 10 μL of sample homogenized in a 50 mM phosphate buffer at pH 7.4. After 30 min of incubation at 27 °C, the formation rate of formazan was measured spectrophotometrically at 570 nm [27] with a Varian Cary 50MPR spectrophotometer (Varian Ltd., Melbourne, Australia). Each sample was tested in triplicate, and the absorbance values were normalized to the total protein amount in the sample.

### 2.6. Acetylcholinesterase (AChE) Activity

Homogenates of heads and thoraces (0.01 mg protein) were incubated in a solution containing 100 mM sodium phosphate buffer (pH 7.4) with 150 mM acetylthiocholine (CAS no. 1866-15-5) and 1 mM DTNB. AChE activity was determined spectrophotometrically at 412 nm using a Varian Cary 50MPR spectrophotometer (Varian Ltd., Melbourne, Australia). The activity was expressed as nanomoles of conjugate formed per minute per milligram of protein [28,29].

#### Protein Quantification

Protein concentration was measured using the Bradford method according to the manufacturer’s instructions (Labtest, Brazil, cat. no. 99-250). The assay measures the interaction between the protein and the reagent, with absorbance readings taken at 545 nm [30].

### 2.7. Statistical Analysis

Data are shown as the mean ± SEM, with N indicating the number of female flies per group in each experiment. Statistical analyses were conducted using GraphPad Prism version 8.0 software (San Diego, CA, USA). To evaluate the significance of differences between groups, *t*-tests with Bonferroni’s post hoc tests were used for untreated and treated flies. Differences were considered significant at *p* < 0.05.

## 3. Results

### 3.1. Cholesterol Levels

**Figure 1:**
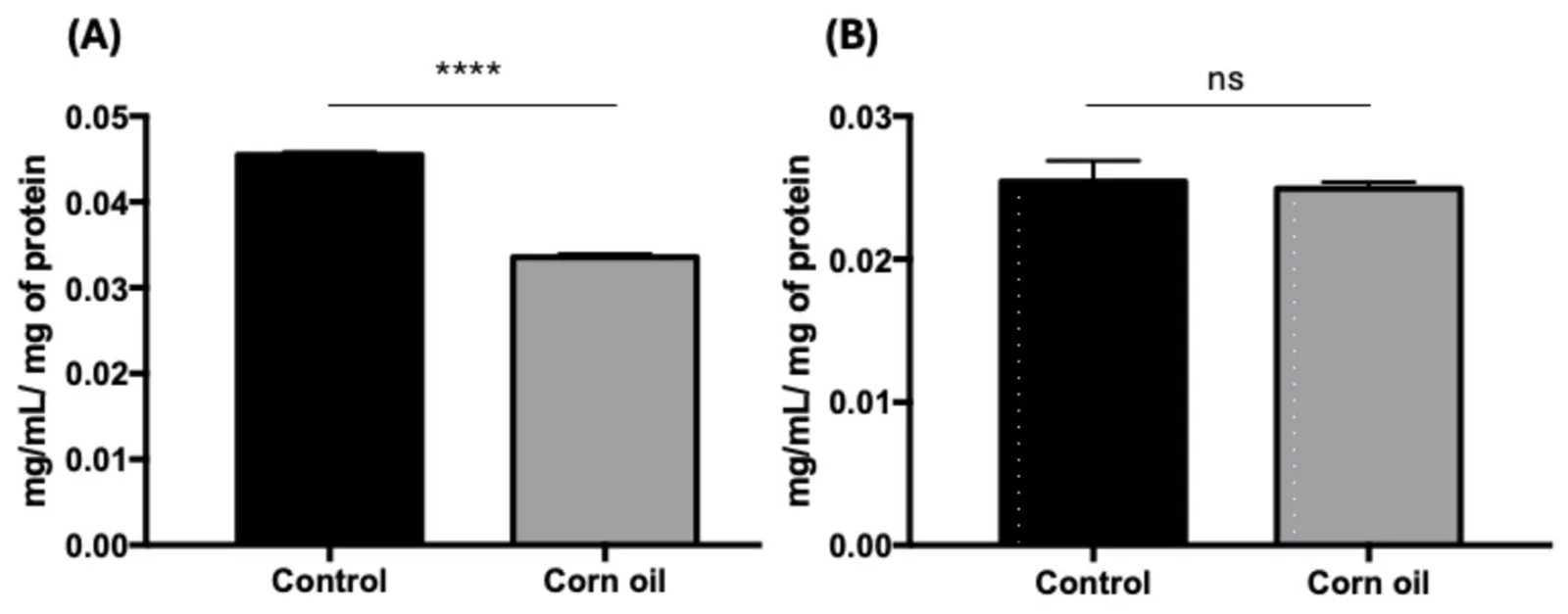
Cholesterol levels in *D. melanogaster*, showing (**A**) head and (**B**) thorax from female APP-β42-expressing flies fed either a standard diet or a corn oil-supplemented diet. Data represent three independent biological replicates (n = 3), each consisting of pooled tissues from 100 females. The values represent the mean ± SEM (unpaired *t*-test) of three experiments. Differences were statistically significant at **** *p* < 0.0001, and ‘ns’ indicating *p* > 0.05.

Flies expressing human APP and β_42_ fragments were fed a standard diet, leading to a significant increase in head cholesterol levels (*p* < 0.001) (Figure 1A). In contrast, there were no notable differences in cholesterol levels in the thorax (Figure 1B).

#### 3.1.1. Catalase Activity and Hydrogen Peroxide Production

**Figure 2:**
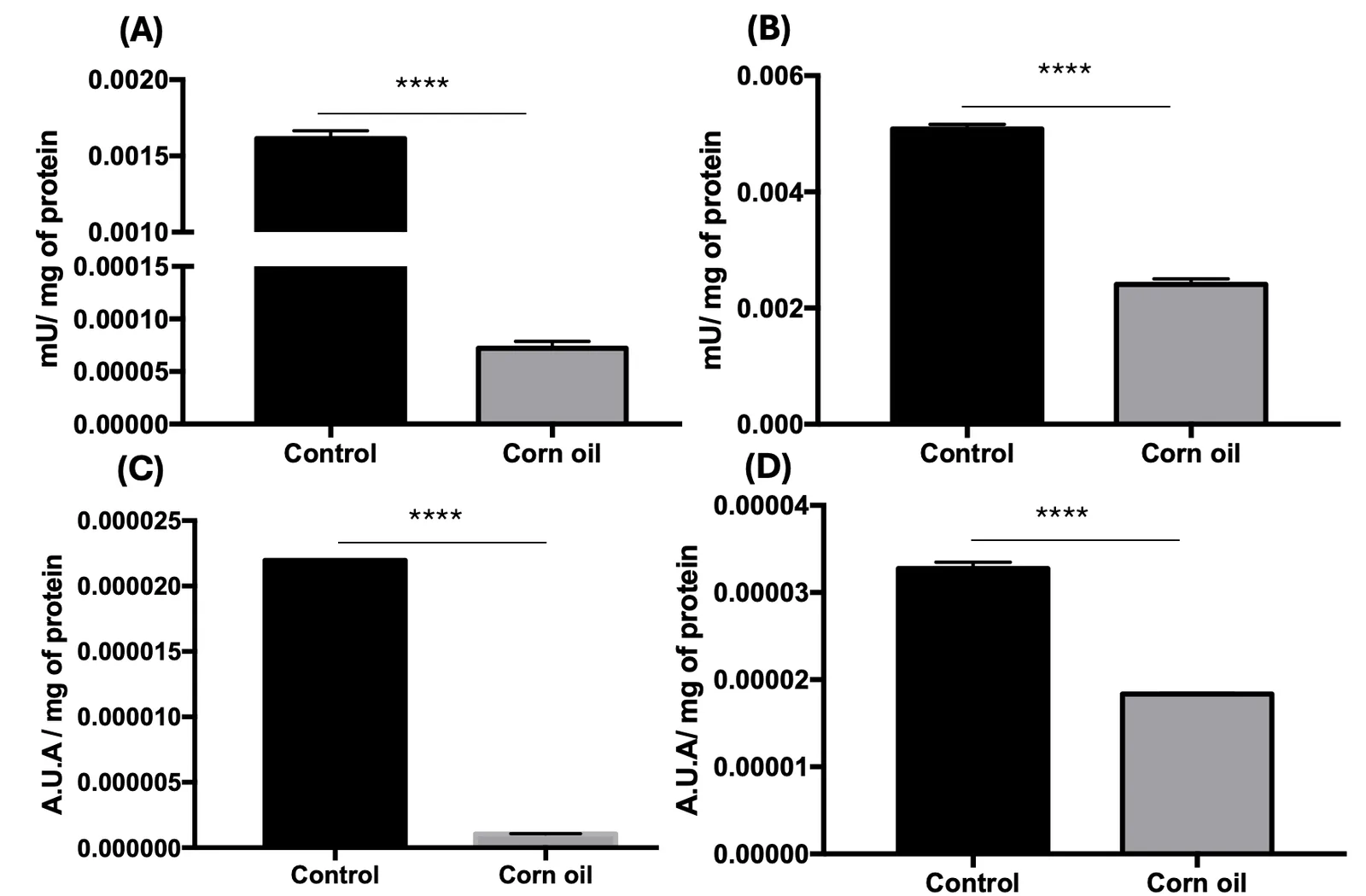
Catalase activity and H_2_O_2_ production in (**A**,**C**) heads (n = 3) and (**B**,**D**) thoraces (n = 3) of *D. melanogaster* fed a diet supplemented with corn oil. The values represent the mean ± SEM (unpaired *t*-test) of three experiments. The results were statistically significant at **** *p* < 0.0001.

Ingestion of corn oil significantly decreased catalase activity in the heads (*p* < 0.0001) (Figure 2A) and thoracic muscles (*p* < 0.0001) (Figure 2B). Additionally, hydrogen peroxide (H_2_O_2_) levels were reduced in the heads (*p* < 0.0001) (Figure 2C) and thoraces (*p* < 0.0001) (Figure 2D) of these flies.

#### 3.1.2. Reduced Glutathione Levels

**Figure 3:**
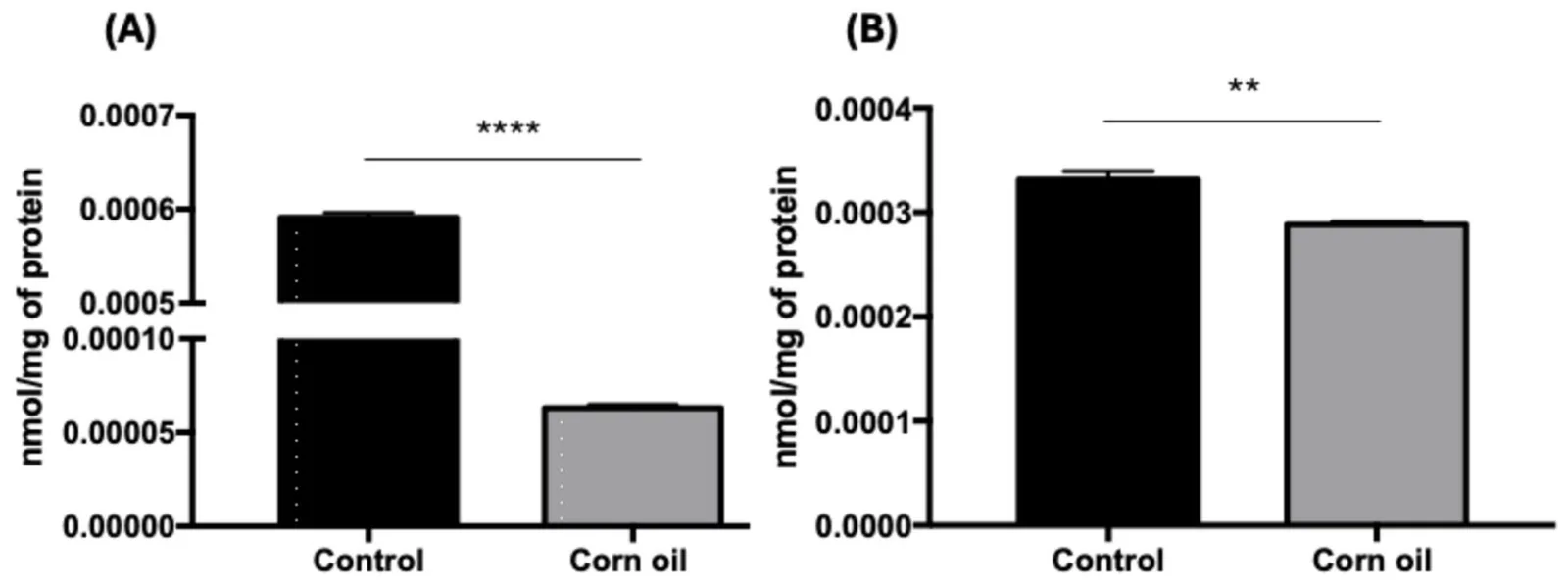
GSH concentration in (**A**) heads (n = 3) and (**B**) thoraces (n = 3) of *Drosophila melanogaster* fed a diet supplemented with corn oil. The values represent the mean ± SEM (unpaired *t*-test) of three experiments. The results were statistically significant at ** *p* < 0.01 and **** *p* < 0.0001.

Mutant flies fed corn oil exhibited decreased levels of reduced glutathione in their heads (*p* < 0.0001) (Figure 3A) and muscles (*p* < 0.01) (Figure 3B).

### 3.2. Formazan Generation and Citrate Synthase (CS) Activity

**Figure 4:**
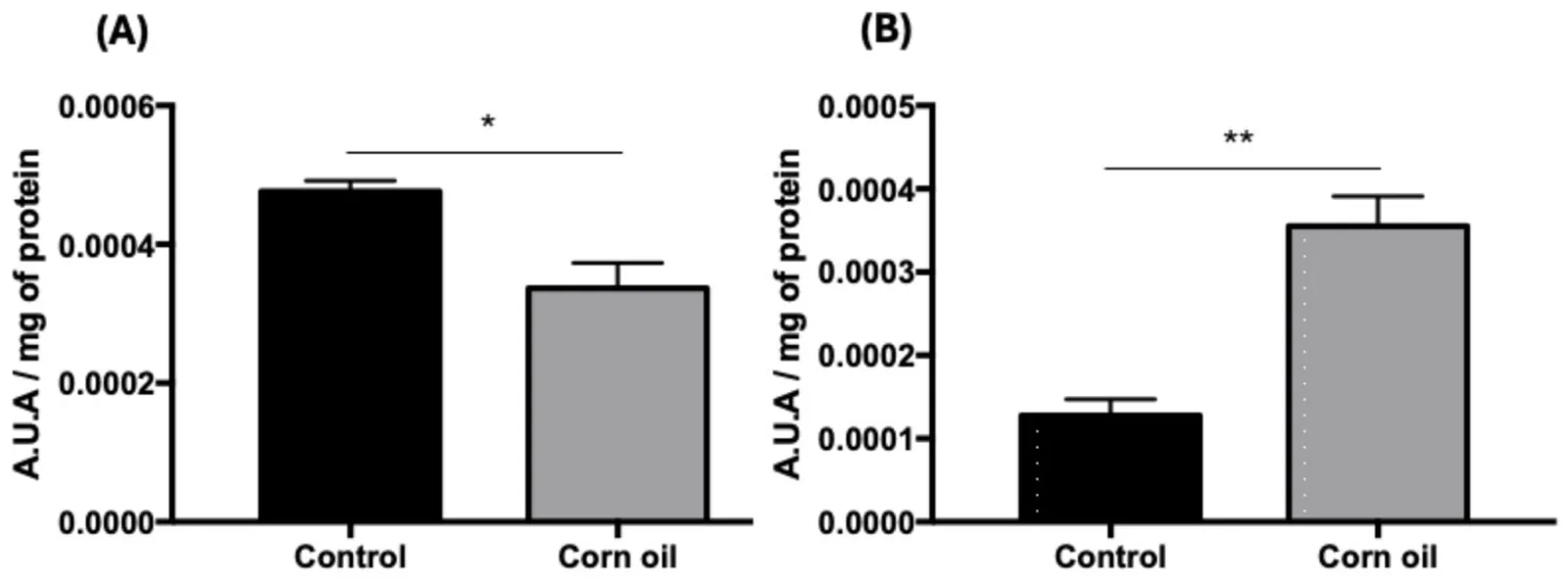
Formazan production in the (**A**) heads (n = 3) and (**B**) thoraces (n = 3) of flies fed a standard diet or a diet supplemented with corn oil. The values represent the mean ± SEM (unpaired *t*-test) of three experiments. The results were statistically significant at * *p* < 0.05 and ** *p* < 0.01.

**Figure 5:**
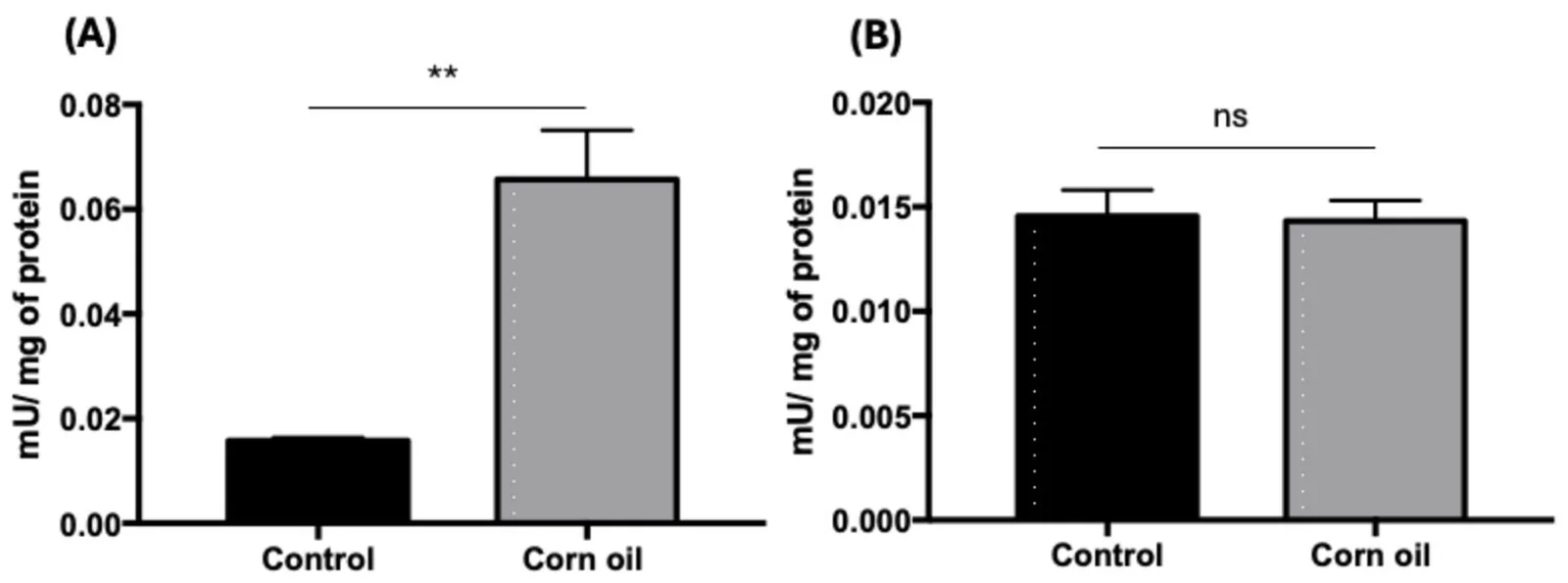
CS activity in the (**A**) head (n = 3) and (**B**) thorax (n = 3) of *D. melanogaster*. The values represent the mean ± SEM (unpaired *t*-test) of three experiments. The results were statistically significant at ** *p* < 0.01; ‘ns’ indicating *p* > 0.05.

Mutant flies fed corn oil exhibited a decrease in formazan production (*p* < 0.01) (Figure 4A) in the head and a higher concentration in the thoracic muscle (*p* < 0.01) (Figure 4B). The CS activity increased (*p* < 0.01) in the heads of flies fed corn oil (Figure 5A). However, in the thoracic muscle, ingestion of corn oil showed no significant change in CS activity (Figure 5B).

#### Lactate Levels

**Figure 6:**
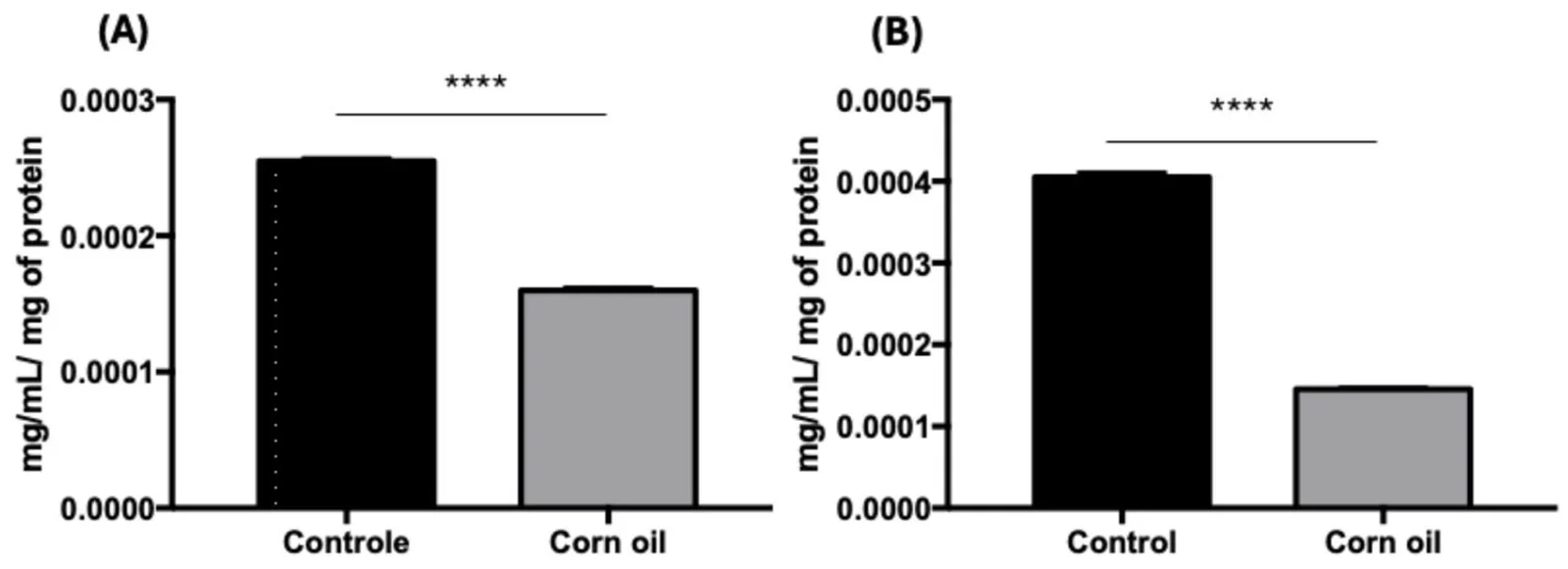
Lactate concentration in the (**A**) heads (n = 3) and (**B**) thoraces (n = 3) of flies fed a diet supplemented with corn oil. The values represent the mean ± SEM (unpaired *t*-test) of three experiments. The results were considered statistically significant at **** *p* < 0.0001.

Lactate levels decreased in the heads (Figure 6A) (*p* < 0.0001) and thoraces (Figure 6B) (*p* < 0.001) after flies were fed corn oil for 5 days.

### 3.3. Acetylcholinesterase (AChE) Activity

**Figure 7:**
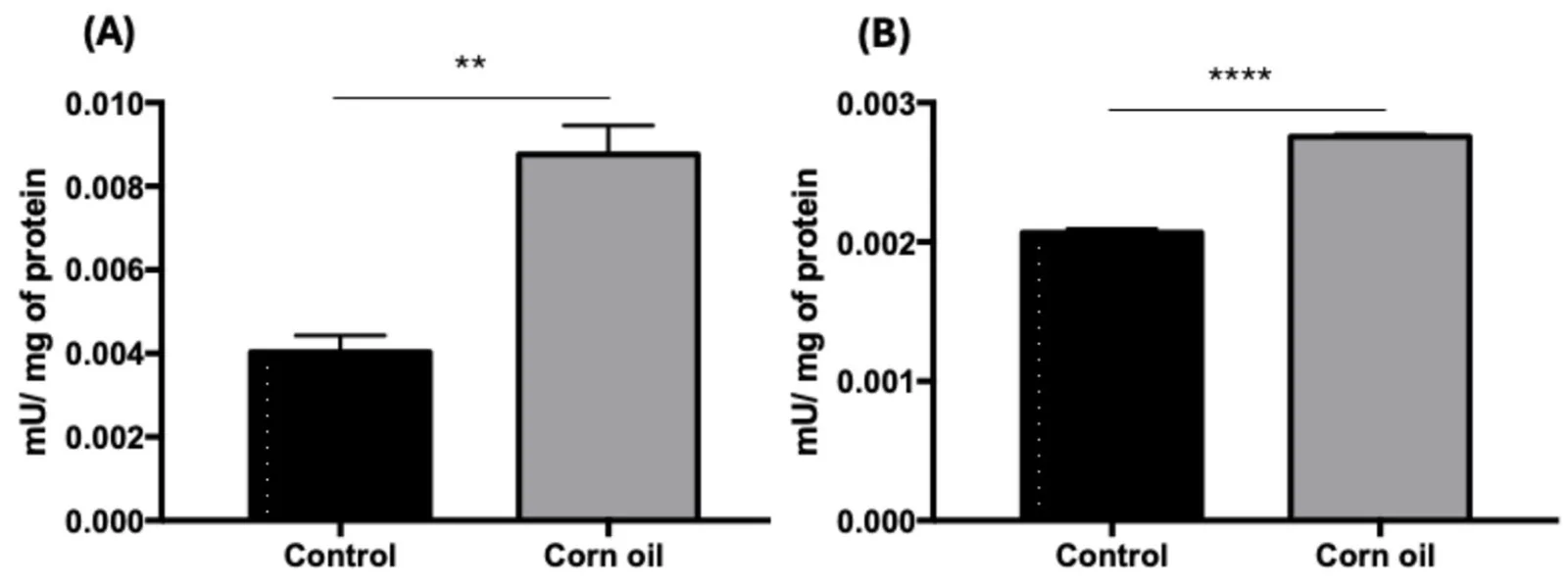
AChE activity in the (**A**) heads (n = 3) and (**B**) thoraces (n = 3) of mutant *Drosophila melanogaster* fed a diet supplemented with corn oil. The values represent the mean ± SEM (unpaired *t*-test) of three experiments. The results were statistically significant at ** *p* < 0.01 and **** *p* < 0.0001.

Ingestion of corn oil resulted in a significant rise in acetylcholinesterase (AChE) activity in the heads (*p* < 0.01) (Figure 7A) and thoracic muscles (*p* < 0.0001) (Figure 7B).

## 4. Discussion

Polyunsaturated fatty acids (PUFAs) are essential nutrients that must be obtained through diet because the human body cannot synthesize them [31]. They play important roles in maintaining cell membrane structure, regulating blood pressure and blood clotting, supporting immune function, facilitating the absorption of fat-soluble vitamins, and modulating inflammatory processes [31]. PUFAs have also been associated with improved cardiovascular health [32,33], cognitive and neuroprotective effects [34], and anti-inflammatory properties [35].

Corn oil is rich in linoleic acid (LA), an essential omega-6 PUFA that contributes to normal growth and development [36]. Previous studies have shown that LA can influence several cellular processes, including oxidative stress, inflammation, mitochondrial function, and endoplasmic reticulum stress, which may contribute to organ dysfunction under specific experimental conditions [36,37]. However, despite LA being essential for health, a higher intake can lead to the formation of oxidized linoleic acid metabolites (OXLAMs). OXLAMs can cause mitochondrial damage and have been associated with several diseases like Alzheimer’s and cancer [38].

The consumption of unsaturated fatty acids can directly affect the fatty acid profile of *Drosophila melanogaster* tissues, modify phospholipid composition, and thereby influence cell membrane fluidity and function [39]. Hunter-Manseau and colleagues (2024) [40], in their study on *Drosophila melanogaster,* demonstrated that the consumption of a high-fat diet without a prior fasting period resulted in decreased CS activity and complex IV, consequently causing mitochondrial impairment by drastically reducing the respiration rate.

Nevertheless, it is important to emphasize that the present study evaluated supplementation with whole corn oil rather than isolated PUFAs or linoleic acid. Therefore, the biochemical effects observed here cannot be attributed specifically to PUFAs or to LA. Instead, they should be interpreted as effects of corn oil supplementation, with its PUFA content representing one of several components potentially contributing to the observed responses.

Qualitative observations from the SMURF assay suggested differences in intestinal barrier integrity between the dietary groups. In this assay, the presence of Brilliant Blue FCF outside the gastrointestinal tract indicates a loss of intestinal barrier integrity and leakage of intestinal contents into systemic tissues [20]. Flies maintained on the corn oil-supplemented diet appeared to show less extensive dye leakage than those maintained on the standard diet (Figure S2). However, because this observation was not quantitatively assessed, it should be interpreted as supportive qualitative evidence rather than as a demonstration of a protective effect of corn oil on intestinal permeability.

Oxidative stress involves various free radicals and reactive molecules derived from molecular oxygen, nitrogen, and sulfur [41]. When these molecules reduce electron availability, they produce superoxide anions (O_2_^•−^), which can react with other cellular components to form more reactive species, such as hydrogen peroxide (H_2_O_2_) and hydroxyl radicals (^•^OH) [42]. Although these reactive species are natural byproducts of mitochondrial respiration, excessive generation can cause significant damage to lipids, proteins, and DNA [43]. Cells counteract this through an antioxidant system that includes enzymes like catalase and molecules such as reduced glutathione (GSH), which help prevent substantial cellular damage [44]. GSH particularly supports catalase by detoxifying residual H_2_O_2_ and other peroxides produced during oxidative stress [44].

In Alzheimer’s disease (AD), Aβ disrupts normal mitochondrial function, resulting in alterations in oxidative phosphorylation. This involves a reduction in electron transfer efficiency, which leads to increased production of reactive oxygen species (ROS), primarily at complexes I and III. Additionally, the Aβ peptide not only promotes the generation of ROS at the mitochondrial level but also simultaneously inhibits the removal of ROS [45,46]. Our results showed that mutant flies exposed to AD produced high levels of H_2_O_2_, suggesting that oxidative stress was induced. However, supplementation with corn oil was effective in mitigating the damage caused by oxidative stress, as it enhanced the antioxidant capacity of PUFAs or improved mitochondrial parameters [18].

Additionally, we used the pyrogallol and 3-(4,5-dimethylthiazol-2-yl)-2,5-diphenyltetrazolium bromide (MTT) assay to assess the samples’ antioxidant capacity within a superoxide-generating system [47]. Pyrogallol’s self-oxidation produces superoxide, which reduces MTT to formazan through electron transfer by succinate dehydrogenase or mitochondrial complex II. Our findings show that *D. melanogaster* fed corn oil exhibited less formazan formation in the head region, indicating enhanced mitochondrial complex II activity and stronger local antioxidant defense as described in wild-type flies [18]. However, ingestion of corn oil demonstrated a higher formazan level in the thoraces, suggesting a reduction in succinate dehydrogenase activity. Previous studies have induced oxidative stress in *D. melanogaster* larvae and adults using H_2_O_2_, demonstrating that dietary antioxidant supplements decreased H_2_O_2_ levels, boosted antioxidant enzyme activity, and increased survival rates. Higher CAT activity and GSH levels reflected an adaptive response to oxidative stress [48].

The imbalance in cholesterol production contributes to the production and accumulation of β-amyloid peptides (Aβ) and increases β- and γ-secretase activities, favoring the amyloidogenic pathway [49]. Additionally, a reduction in membrane cholesterol displaces the APP protein and secretase activities, favoring the non-amyloidogenic pathway [50,51]. Flies fed corn oil (rich in linoleic acid) showed a reduction in cholesterol in the head, indicating a potential health benefit of the non-amyloidogenic pathway.

Membrane fluidity is affected by cholesterol [52], which can hinder biomolecule transport and enzymatic activity, such as that of acetylcholinesterase (AChE), an enzyme embedded in the membrane responsible for regulating neural signaling [53]. Studies indicate that higher membrane fluidity may affect AChE activity, aiding in the retention of acetylcholine in the synaptic cleft [54,55]. Several studies also demonstrate that PUFAs can enhance membrane fluidity and impact AChE activity, either increasing or decreasing it [55,56,57]. Our results showed increased AChE activity in flies consuming the corn oil-supplemented diet. Changes in redox status, mitochondrial metabolism, and membrane lipid composition may contribute to alterations in AChE activity [58,59]. However, because increased AChE activity may enhance acetylcholine hydrolysis, this response should not be considered inherently beneficial in the context of AD. Rather, the present findings suggest that corn oil supplementation modulates cholinergic metabolism. Finally, our results indicate the preservation of mitochondrial function, accompanied by a reduction in oxidative stress, which may be a cellular adaptation. This suggests that even with mitochondrial impairment, the cells were able to mitigate oxidative stress in flies fed corn oil.

Our work yielded interesting results regarding linoleic acid (LA) supplementation in Drosophila melanogaster genetically modified to model Alzheimer’s disease. However, to further investigate the impact (whether beneficial or detrimental) of LA on disease progression, future complementary experiments could be conducted—such as evaluating the immune response involving the IMD (Immune Deficiency) pathway and NF-κB (Nuclear Factor kappa-light-chain-enhancer of activated B cells). Also, a study demonstrated that high-fat diets can alter the gut microbiota in *Drosophila*, triggering a systemic inflammatory state that affects the animals [60]. The exacerbation of inflammatory processes, such as the c-Jun N-terminal kinase (JNK) pathway, can accelerate neurodegeneration in mutant Drosophila expressing APP-β42, thereby increasing mortality among the modified animals [61]. JNK, in turn, signals the inflammatory cascade by activating Eiger and its receptor, Wengen; Eiger is a homolog of the mammalian tumor necrosis factor-alpha (TNFα) superfamily and, as in mammals, plays a role in the development and regulation of immunity in Drosophila melanogaster [61,62]. Therefore, evaluating the IMD/NF-κB and JNK inflammatory pathways—combined with the biochemical results obtained in the present study—will provide a more reliable assessment of the impact of LA supplementation in *Drosophila* models of Alzheimer’s disease, thereby offering a better understanding of how high-fat diets affect neurodegenerative processes.

## 5. Conclusions

Corn oil supplementation in female flies expressing APP-β42 induced tissue-dependent changes in biochemical, oxidative stress-related, mitochondrial, and neurochemical parameters. The treatment was associated with reduced H_2_O_2_ levels, changes in GSH levels, increased citrate synthase activity in head tissue without significant changes in thoracic tissue, and increased AChE activity. These findings indicate that corn oil supplementation modulates redox, mitochondrial, and cholinergic-related parameters in this experimental model. However, the observed changes should not be interpreted as a uniform enhancement of antioxidant defenses or as direct evidence of a neuroprotective effect. Further studies, including quantitative assessment of AD-related phenotypes, are needed to clarify the biological significance of these effects.

## Data Availability

The data that support the findings of this study are available from the corresponding author upon request.

## Supplementary Materials

The following supporting information can be downloaded at: https://www.mdpi.com/article/10.3390/neurolint18090176/s1, Figure S1: (A) Head of *Drosophila melanogaster* and the analyzed region in the eye. Confocal microscopy in the heads of (B) GMR-Gal4; UAS-APP-β_42_ flies fed a standard diet, and (C) GMR-Gal4; UAS-APP-β_42_ flies fed a standard diet supplemented with corn oil and stained with Congo Red; Figure S2: Permeability of *Drosophila melanogaster* gut (Smurf test). (A) GMR-Gal4; UAS-APP-β_42_ flies fed with standard diet; (B) GMR-Gal4; UAS-APP-β_42_ flies fed with standard diet supplemented with corn oil.

## Abbreviations

The following abbreviations are used in this manuscript:

APP-β_42_	Amyloid precursor protein β42
CS	Citrate synthase
AD	Alzheimer’s disease
Aβ	β-Amyloid peptide plaques
NMDA	N-Methyl-D-aspartate
ARIA	Amyloid-related imaging abnormalities
BDSC	Bloomington Drosophila Stock Center
UAS	Upstream activation sequence
GMR-Gal4	Glass multimer reports Gal4 driver
GSH	Reduced glutathione
CAT	Catalase
H_2_O_2_	Hydrogen peroxide
FOX	Ferrous oxidation with xylenol orange
DTNB	Dithiobis-2-nitrobenzoic acid
AChE	Acetylcholinesterase
PUFAs	Polyunsaturated fatty acids
LA	Linoleic acid
OXLAMs	Oxidized linoleic acid metabolites
O_2_•^−^	Superoxide anions
•OH	Hydroxyl radicals
ROS	Reactive oxygen species
MTT	3-(4,5-Dimethylthiazol-2-yl)-2,5-diphenyltetrazolium bromide
