# Supplementary material for: Impact of Corn Oil on Biochemical and Neurochemical Markers in an Animal Model of Alzheimer’s Disease
Source: Neurol Int. 2026 Sep 17;18(9):176. doi: 10.3390/neurolint18090176 (PMC13610347; doi:10.3390/neurolint18090176)
Supplement: Supplementary file 1 [file neurolint-18-00176-s001.zip › neurolint-4493174-supplementary.pdf]

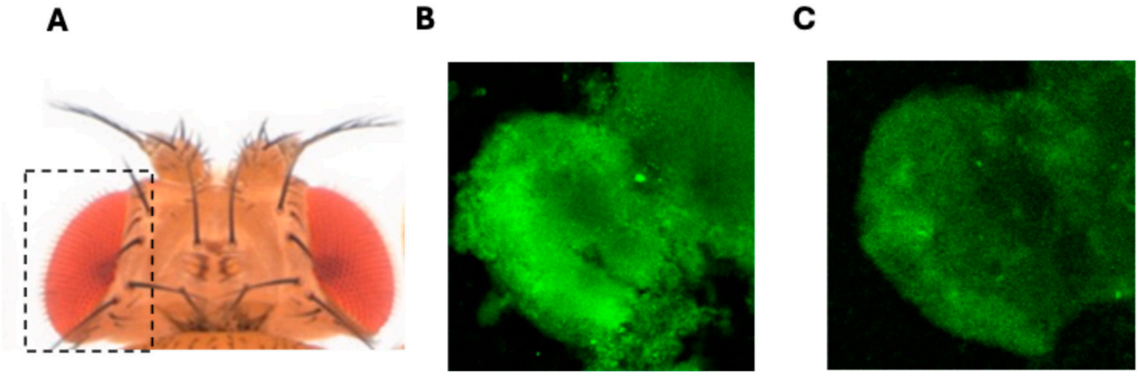

Figure S1. (A) Head of *Drosophila melanogaster* and the analyzed region in the eye. Confocal microscopy in the heads of (B) GMR-Gal4; UAS-APP-β_{42} flies fed a standard diet, and (C) GMR-Gal4; UAS-APP-β_{42} flies fed a standard diet supplemented with corn oil and stained with Congo Red.

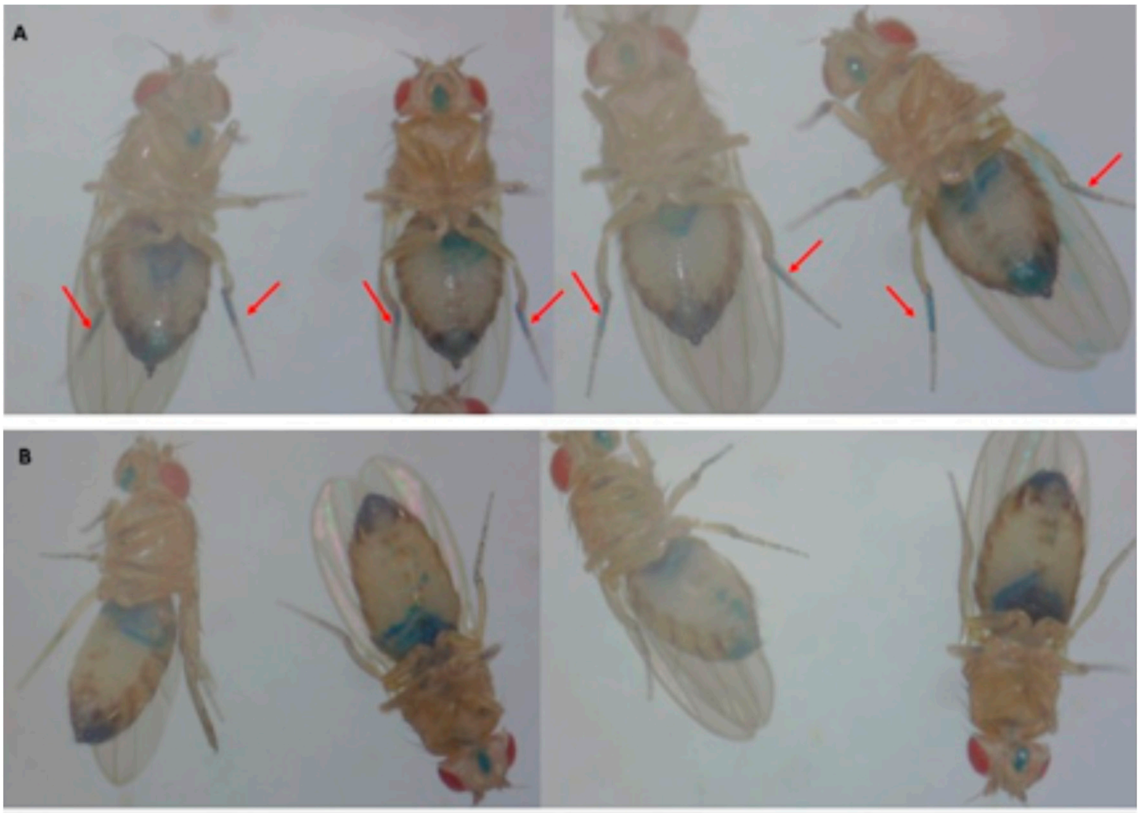

Figure S2. Permeability of *Drosophila melanogaster* gut (Smurf test). (A) GMR-Gal4; UAS-APP-β_{42} flies fed with standard diet; (B) GMR-Gal4; UAS-APP-β_{42} flies fed with standard diet supplemented with corn oil.
